# Persistence and fading of the cognitive and socio-emotional benefits of preschool education in a low-resource setting: Group differences and dose-dependent associations in longitudinal data from Vietnam

**DOI:** 10.3389/fpsyg.2023.1065572

**Published:** 2023-02-07

**Authors:** Phuong Thi Thu Dinh, Julie Ann Robinson

**Affiliations:** ^1^College of Education, Hue University, Hue, Vietnam; ^2^Kinder in Wien, Vienna, Austria; ^3^College of Education, Psychology and Social Work, Flinders University, Adelaide, SA, Australia

**Keywords:** preschool education, fade-out, low- and middle-income countries, life satisfaction, self-concept, relationships, vocabulary, mathematics

## Abstract

**Introduction:**

Four analytic approaches examined the effectiveness of preschool education in Vietnam, which provides a context in which national curricula and teaching standards for preschools and schools, high levels of preschool attendance, and fee subsidies for disadvantaged children, limit the heterogeneity in children’s experiences that often obscure the outcomes associated with preschool attendance.

**Methods:**

The Young Lives Study provided longitudinal data on children’s receptive vocabulary, mathematics, and life satisfaction at 5, 8, 12, and 15 years of age, and on their self-concept and relationships at 12 and 15 years.

**Results:**

The first analysis found that children who attended preschool (*n* = 1,562 at 5 years of age) had larger vocabularies at 5, 8, 12, and 15 years, greater mathematics knowledge at 5, 8, and 12 years, and higher life satisfaction at 5 and 12 years of age than the small number of children who did not attend preschool (*n* = 164 at 5 years of age). The second, found that the dose of preschool education (hours per week × 4 × months) received by children who attended preschool was positively associated with their receptive vocabulary and mathematics scores at 5, 8, 12, and 15 years of age, and with their life satisfaction at 5 and 15 years of age. Although the magnitude of the effect for vocabulary declined over time, it remained stable for mathematics. The third analysis found that a high dose of preschool education allowed disadvantaged rural children to achieve comparable or better scores than their urban peers for receptive vocabulary at 8, 12, and 15 years, mathematics at 12 years, and life satisfaction at all ages. The final analysis found that even a low dose of preschool education improved rural children’s receptive vocabulary at 5, 8, and 15 years, and their numeracy/mathematics scores at 5, 8, and 12 years.

**Discussion:**

Together, the results suggest that preschool attendance had a small but meaningful positive association with Vietnamese children’s cognitive skills and life satisfaction that persisted for at least 10 years. These findings provide insights into the scale, scope, and longevity of effects that can be achieved from scaled-up preschool programs under resource-constrained conditions.

## 1. Introduction

Recent estimates suggest that in low- and middle-income countries 250 million children under the age of 5 years are at risk of not reaching their developmental potential (Black et al., 2017). This represents 43% of young children in these countries and therefore has profound implications not only for these children’s life opportunities but also for their countries’ economic prosperity. Global recognition of the importance of early childhood development led to the inclusion of a dedicated target in the UN Sustainable Development Goals. Target 4.2 calls on countries to “*ensure that, by 2030, all girls and boys have access to quality early childhood development, care and pre-primary [preschool] education so that they are ready for primary education*” (UN General Assembly, 2015).

To facilitate progress toward this goal, many international development agencies strongly advocate for the introduction of universal preschool education in low- and middle-income countries and have provided funding or other resources to support this. These agencies include Save the Children (Save the Children International, 2017), the United Nations Children’s Fund (Borisova et al., 2020), the United Nations Educational, Scientific and Cultural Organization (UNESCO, 2006), and the World Bank (Bendini and Devercelli, 2022). The goal of such advocacy is to overcome chronic poor academic achievement in these countries, especially among children in rural areas (World Bank, UNESCO, and UNICEF, 2021; United Nations Children’s Fund, 2022). The focus on preschool education is partially motivated by evidence that when children raised in poverty in high-income countries receive high quality, custom-designed school readiness programs, these have a long-lasting positive influence on their social, educational, and economic outcomes, and trans-generational benefits for their families and communities (e.g., Heckman and Karapakula, 2019; McCoy et al., 2019; Pages et al., 2022).

Less-intensive generic public preschool education programs in high-income countries can also support small to moderate positive gains in cognitive outcomes in the short (Magnuson and Waldfogel, 2005), intermediate (Davies and Brember, 1997; Sylva et al., 2004; Havnes and Mogstad, 2011; Ladd et al., 2014), and longer term (Barnett, 1998; Goodman and Sianesi, 2005; Apps et al., 2013; Sisco et al., 2014). The benefits of preschool education are often especially noticeable for children from disadvantaged socio-economic backgrounds (Sylva et al., 2004; Havnes and Mogstad, 2011; Apps et al., 2013).

However, it may be unrealistic to expect that benefits of the magnitude reported from preschool education in high-income countries can be replicated in low- and middle-income countries. In high-income countries, the preschool programs that provide the strongest and most durable benefit differ substantially from the programs available in most low- and middle-income countries, which are characterized by poor quality in both structural (e.g., classroom, hygiene, and sanitation facilities; staff:student ratios) and process dimensions (e.g., pedagogy; discipline practices; e.g., Aboud, 2006; Campos et al., 2011). Despite this, some research suggests that participation in the generic preschool programs offered in diverse low- and middle-income countries may confer benefits. These include better performance in reading, writing, and oral mathematics in Bangladesh (Aboud and Hossain, 2011), Spanish and mathematics performance in Argentina (Berlinski et al., 2009), and receptive vocabulary and mathematics performance in Ethiopia (Woldehanna, 2016). Preschool attendance is also associated with higher levels of school enrolment and/or progress through grades in Mozambique, Zambia, Uganda, India, and Nepal (Martinez et al., 2012; Zuilkowski et al., 2012; Hazarika and Viren, 2013; Jaganath et al., 2015; Nyeko et al., 2015).

However, there are two important caveats concerning these findings. First, very few studies include sub-national analyses, so it remains unclear whether preschool education contributes to the achievement of these countries’ social justice goals, for example, by reducing chronic rural educational disadvantage (e.g., Conference of Ministers of Education of French-Speaking Countries, 2015), or whether it primarily confers additional benefits on the most advantaged children. Second, findings suggest that children in low- and middle-income countries achieve very few learning gains from attending some preschool programs (or from school attendance; Gove et al., 2018). For example, in Malawi, even after attending preschool and completing four grades of primary education, children could only identify a mean of 1.29 letters of the alphabet per minute (Pouezevara et al., 2012). Indeed, it is possible that poor quality preschool education may undermine, rather than promote, the development of young children’s cognitive and socio-emotional skills (Cascio, 2015).

Moreover, even in high-income countries, many studies report that most of the differences between children who have and have not attended preschool have disappeared after only 1 or 2 years of schooling (e.g., Puma et al., 2012; Bassok et al., 2015; Li et al., 2020; Burchinal et al., 2022). The strength and pace of this “fade-out” phenomenon (Abenavoli, 2019) is not uniform. It differs across outcomes (Magnuson et al., 2007; Li et al., 2020) and students’ backgrounds (e.g., poverty: Magnuson et al., 2007). Most attempts to identify the factors that contribute to, and limit, the fade-out of benefits from preschool education in high-income countries focus on process factors (e.g., Vitiello et al., 2020; Burchinal et al., 2022) or on dose and delay (e.g., Reynolds et al., 2011; Li et al., 2020). For example, Burchinal et al. (2022) concluded that fade-out is influenced by the types of skills on which the preschool curriculum focuses. In contrast, Li et al. (2020) found that fade-out was influenced by the age at which children started preschool, the duration of their preschool program, and the time between the end of preschool and the assessment of outcomes.

It is currently unclear whether the pace of fade-out or the factors that influence it are similar in low- and middle-income countries. Well-designed evaluations of the long-term benefits of “universal” preschool education programs in low- and middle-income countries are very rare. Moreover, most available research findings are difficult to interpret for six reasons. First, research has often evaluated multi-faceted early childhood interventions in which preschool attendance is confounded with nutritional support (e.g., Raine et al., 2001; Chen et al., 2019) and/or parenting programs (e.g., Martinez et al., 2017). Second, it is often impossible to distinguish the outcomes from education-focused programs for children between 3 and 6 years of age from programs in “early childhood development and care,” such as daycare, that are offered between birth and 6 years. This can be the result of the limitations of measures (e.g., Berlinski et al., 2008; Hazarika and Viren, 2013; Gong et al., 2016; Zhang, 2017; Gove et al., 2018; Shafiq et al., 2018; Yang, 2021) or the authors’ explicit focus on the broader range of programs (e.g., Montie et al., 2006; Baker-Henningham, 2014; Jackson et al., 2019). Third, data are often pooled across preschool programs that differ markedly in content and quality and in the qualifications and pedagogical practices of those who deliver the programs, despite evidence that these factors influence participants’ outcomes (e.g., Mwaura et al., 2008; Rao et al., 2012). Fourth, there is also often heterogeneity in the content and quality of the schooling children receive between the completion of their preschool program and the assessment of their long-term gains from that program. Fifth, many studies evaluate demonstration projects (e.g., Mwaura et al., 2008; Martinez et al., 2012). These may provide limited information about the likely benefits of a universal preschool program because many are not feasible to deliver “at scale” in low-resource settings. In addition, the efficacy of demonstration projects is substantially greater than their effectiveness under the implementation constraints that apply when a program is “scaled up” (e.g., Grantham-McGregor and Smith, 2016; Supplee et al., 2022; Sutherland et al., 2022). Sixth, in the majority of studies, preschool attendance is biased in ways that advantage children who attend. This bias applies in two contexts. In many studies preschool education is not widely accessible, and where it is accessible fewer than 50% of eligible children attend (e.g., Martinez et al., 2012; Gong et al., 2016; Woldehanna, 2016). In this context, preschool attendance is the result of positive selection by parents who have the motivation to invest in their children’s early education and the means to do so. Such motivation and means have been found to be loosely associated with specific demographic characteristics, such as higher levels of parental education and income, that are also associated with positive child development and higher future academic performance (e.g., Martinez et al., 2012; Rao et al., 2012; Gong et al., 2016; Woldehanna, 2016). Conversely, in contexts in low- and middle-income countries in which there are very high levels of preschool enrolment, it can be predicted that parents who “opt out” of preschool education for their child also possess distinctive motivational and demographic characteristics. In both contexts, biases in preschool attendance can result in the identification of spurious differences when the outcomes of children who did and did not attend preschool are compared. Most studies attempt to address this challenge by including several demographic characteristics as covariates in their analyses (e.g., Aboud and Hossain, 2011; Rao et al., 2012) or using propensity score matching techniques (e.g., Gong et al., 2016). However, these may remove important nuances in the data without eliminating the unobserved group differences in child characteristics and parental motivations, values and behaviors that influence preschool attendance. A range of sophisticated statistical techniques and machine learning approaches have also attempted to overcome this challenge (Berlinski et al., 2008, 2009; Nguyen and Goutte, 2022).

One strategy that preserves nuances in the data is to focus on children who attended preschool and to examine the relationship between the dose of preschool education they received and their outcomes. Studies of dose-dependent effects of preschool education in low- and middle-income countries are extremely rare. However, some studies of multi-faceted programs with a preschool component, and studies in which preschool education is included as one form of early child development and care, have collected data on the duration of attendance or the age at which children commenced preschool. Effects are often greater for children who attended these programs for longer (e.g., Martinez et al., 2012) although this pattern is not observed for all outcomes (e.g., Gong et al., 2016; Zhang, 2017) or for all programs, some of which appear to have a ‘threshold dose’, above which no additional benefit is gained (Jackson et al., 2019). The extent to which these findings can be applied to preschool education in particular, remains unclear. Both dose-dependent and dose-threshold relationships are plausible. For example, there may be a threshold effect when large mixed-age classes are led by teachers with limited training and where few teaching and learning resources are available. In such contexts, learning activities and pedagogical approaches that are inclusive of younger children may be repeated over time.

Ideally, research that examines long-term dose-dependent relationships between preschool attendance and children’s outcomes in low- and middle-income countries would be conducted in contexts in which there is “universal” preschool attendance; children from different geographic and socio-economic backgrounds complete the same national preschool curriculum that is delivered by teachers with similar training; and the primary and secondary schools that children subsequently attend also follow national curricula and are staffed by teachers with similar levels of training. Vietnam provides a context that approximates these conditions.

Vietnam has a long history of preschool education (Thao and Boyd, 2014). Thus, preschool teacher training programs and administrative infrastructure in Vietnam predate the recent focus on early childhood by international development agencies. Children are eligible to enroll in preschool (Trường Mẫu giáo) between their third birthday and school entry at 6 years of age. Parents can choose to enroll their children in full-day (from 7 am to 4–5 pm) or half-day (from 7 am to 12 pm) programs (Hoang, 2019). In 2003, the Vietnamese government committed to a plan to have all 5-year-old children attend a preschool program, and in 2009 it became compulsory for all 5-year-old children to complete 1 year of preschool education (Abbott et al., 2019). The curricula aim to develop the physical, emotional, mental, cognitive and social development of children, and prepare them for school (Hoang, 2019). However, because preschools articulate with a system of child care centers catering to children aged from 0 to 3 years (nhà trẻ), and the same center may offer both programs (Trường mẫu giáo), some parents use enrolment in preschool to continue to access child-minding services while they are at work (Abbott et al., 2019). In 2004, the *Law on Child Protection, Care and Education* was adopted. This required the government to provide exemptions and subsidies for preschool fees for disadvantaged children to ensure social justice. Since 2006, the government’s support for the daily operation of preschools has focused on deprived areas. In other areas preschools are now organized by parents and communities or provided by the private sector. The national Ministry of Education and Training continues to be responsible for regulating the quality of preschools, standardizing the curriculum, training teachers and applying national standards for preschool teachers (Boyd and Thao, 2017; Abbott et al., 2019). Until recently, the most common qualification for preschool teachers was a professional secondary school diploma (trung cấp sư phạm) in early childhood education, normally awarded by a specialist teacher-training school. The Ministry provides teaching guidelines for the curriculum, and its local offices supervise its implementation (Vu, 2021). All primary and secondary schools also follow a standard national curriculum; and the government has set minimum training standards for teachers at all levels of the education system (Griffin et al., 2006; Nguyen and Pham, 2022). Thus, in the Vietnamese context, attendance at preschool is not dependent on parents’ motivation to invest in their children’s education or other relevant values, and limited heterogeneity in the content and quality of preschool education and children’s subsequent schooling increase the clarity with which any long-term effects of preschool education can be observed.

Even greater clarity would be achieved by limiting errors in data about children’s preschool attendance through the use of contemporaneous measures rather than retrospective reports collected many years later (e.g., Berlinski et al., 2008, 2009; Gong et al., 2016) and by using a long-term longitudinal research design to avoid the confounding of child age with cohort differences that is inherent in cross-sectional designs (e.g., Berlinski et al., 2008, 2009). In addition, in contexts in which national curricula and teaching standards apply, it is desirable to eliminate treatment differences introduced when wealthy communities create demand for elite private preschools that use more diverse and higher quality teaching and learning materials and employ teachers with particularly high professional skills. A sample that approximates these ideals is available for Vietnam. The *Young Lives Study* in Vietnam (Young Lives, 2014; Espinoza et al., 2018) collected longitudinal data on children across multiple sites between infancy and 15 years (the age at which compulsory education ends), including data on preschool attendance when children were 5–6 years old. Due to its focus on development among children raised in poverty, it did not recruit children from communities in the highest wealth quartile and recruited only 23% of the sample from the second highest wealth quartile (Nguyen, 2008; Boyden and James, 2014). An added advantage of The Young Lives–Vietnam dataset is that the participants were of preschool age prior to preschool education becoming compulsory, allowing both dose-effect analyses and treatment-comparison group analyses to be conducted.

Only two previous studies have capitalized on these advantages. One study examined children’s receptive vocabulary and knowledge of mathematics when they were 12 years old (Rogers et al., 2019). It compared children who did not attend preschool with children who attended preschool for three different periods of time (less than 1 year, from one to less than 2 years or more than 2 years). The results were unclear. An ordinary least squares regression found no effect for duration of preschool education for either vocabulary or mathematics scores when the criterion for significance was *p* < 0.05. In contrast, a 2-stage least squares regression led the authors to conclude that 1 year in preschool corresponded to an increase of 2.8 standard deviations on the vocabulary test and 1.3 standard deviations on the mathematics test. These estimates are many times greater than the differences between the relevant group means. Although the authors favored the results of the 2-stage least squares regression, the extent to which its underlying assumptions were appropriate remains unclear.

The second study focused on children who attended preschool. It entered 50 potential control variables into an analysis using a double machine learning approach (Chernozhukov et al., 2018) to estimate the benefits of a one-year earlier preschool starting age on children’s outcomes at 8, 12, and 15 years of age (Nguyen and Goutte, 2022). Although the study found statistically significant effects for language and mathematics skills at some ages, these accounted for less than 0.1 standard deviations in all cases. Similarly, although a 1-year earlier preschool starting age was associated with an increase in the highest grade achieved by 15 years of age, the difference (0.047 years) equates to only 17 days. In addition, the interpretation of findings is complicated by a number of distinctive analysis decisions. First, the authors entered the number of extra hours of classes children attended into their machine learning model as a proxy measure of parental investment in their children’s education, even though parent’s motivation to make this specific investment may be influenced by whether their child’s academic skills require remediation. Second, rather than basing their analyses on children’s receptive vocabulary and reading comprehension scores, the authors used these to derive a composite language skills score. Third, rather than using the scores for the two self-concept measures and two relationship quality measures the authors assumed that a single socio-emotional skills variable underpinned all of these measures and their analyses included only the items that a factor analysis identified as being relevant to a one-factor solution. The resulting reduction in the distinctiveness of these four measures may have contributed to the analyses identifying an effect in only one measure at one child age.

By definition, low- and middle-income countries have scarce resources to invest in supporting early child development. The delivery of a universal preschool program imposes substantial infrastructure and personnel requirements and changes in community attitudes and behaviors. In order to make the hard choices required to deploy resources most efficiently, national policymakers and international development agencies need evidence about the long-term benefits of preschool education delivered “at scale” under the constraints that apply in low-resource settings so that these can be compared against the benefits of committing resources to children’s nutrition, health, and basic education. Information is needed about both the magnitude of effects and their persistence over time.

The current study sought to examine the magnitude and persistence of any benefits from Vietnam’s national preschool program using four analytic approaches. First, a treatment-comparison group analysis addressed two research questions:

1. At 5 years of age, what is the magnitude of any difference in vocabulary, numeracy and socio-emotional outcomes between children who did and did not attend preschool?
2. Do any differences between these groups persist at 8, 12, and 15 years of age?

Second, a dose–response analysis of outcomes for children who had attended preschool, addressed two research questions:

1. What is the magnitude of any association between the dose of preschool education that children received and their vocabulary, numeracy and socio-emotional outcomes at 5 years of age?
2. Do any dose-dependent associations persist at 8, 12, and 15 years of age?

The third and fourth analyses investigated preschool education’s potential to ameliorate rural developmental disadvantage, by addressing two additional questions.

1. Do rural children who attend preschool continue to be developmentally disadvantaged compared to their urban peers? If not, what dose range of preschool education is necessary before this disadvantage is no longer observed?
2. Do rural children who receive a low dose of preschool education (i.e., a dose in the range that may be feasible and sustainable in many low- and middle-income countries) have more favorable outcomes than their peers who do not attend preschool?

## 2. Materials and methods

### 2.1. Data source

The sample for this study was drawn from archival data collected for the Younger Vietnamese Cohort in the *Young Lives study*. This is a longitudinal study conducted by the University of Oxford and funded by the UK Department for International Development (Nguyen, 2008). Children in the Younger Cohort were born in 2002 and quantitative data about them has been collected at approximately 3-year intervals. These children were eligible to attend preschool between 3 and 5 years of age, that is, between 2005 and 2007, 4 years before it became mandatory for all 5-year-old children in Vietnam to receive at least 1 year of preschool education (Abbott et al., 2019). The Vietnamese component of the *Young Lives* study has received ethics clearance from the University of Oxford, the Vietnam Union of Science and Technology Association and the Hanoi School of Public Health (Crivello and Morrow, 2021).

### 2.2. Sampling

Participants (*n* = 2,000) were recruited from 31 communes in five provinces in Vietnam: Lao Cai (North-East region), Hung Yen (Red River Delta), Da Nang (Central urban center), Phu Yen (South-Central coast), and Ben Tre (Mekong River Delta). In each of these provinces, the study purposefully recruited two of the poorest communes (total of 15 communes; 48% of the sample), one commune of average wealth (total of nine communes; 29% of the sample) and one commune with above-average wealth (total of seven communes) (23% of the sample; Nguyen, 2008; Boyden and James, 2014). At the commune level, children who were under 1 year of age were randomly selected. Thus, the data are representative of the 2002 birth cohort at each site. However, because the sample is not nationally representative, it is most useful for investigating the longitudinal dynamics of child development.

### 2.3. Participants

The current analysis focused on data collected when the children were 5 (in 2006, *n* = 1,970), 8 (in 2009, *n* = 1,961), 12 (in 2013, *n* = 1,928), and 15 years of age (in 2016, *n* = 1,912). Most children were members of the Kinh ethnic group, and were from families that subscribed to no religious tradition (Table 1). Nearly 40 percent of mothers had a very low level of education (primary school or less). On average, families’ economic circumstances improved during the course of the study. However, children living in rural locations remained at a marked developmental disadvantage (Table 2).

**Table 1:** Demographic characteristics of the sample.

Characteristic	M	(SD)	%
Ethnic group (*n* = 1,719)
Kinh			88.2
H’mong			5.8
Other			6.0
Child’s religious background (*n* = 1,721)
None			92.4
Buddhist			4.3
Christian			2.2
Other			1.1
Mother’s highest level of education (*n* = 1,722)
Primary school or less			37.0
Junior high school (Grades 6–9)			45.1
High school (Grades 10–12)			10.4
Post-secondary education			7.3
Change in family wealth^
1 year (*n* = 1,998)	0.439	(0.225)	
5 years (*n* = 1,735)	0.500	(0.203)	
8 years (*n* = 1,907)	0.608	(0.189)	
12 years (*n* = 1,926)	0.613	(0.134)	
15 years (*n* = 1,939)	0.708	(0.140)	

^ The Wealth Index was calculated from information concerning quality of housing, access to basic services and ownership of consumer durables (Young Lives, 2002). Scores can range between 0 and 1, with higher scores indicating higher wealth.

**Table 2:** Urban-rural differences when children were 5 years of age in variables associated with impaired child development.

	Urban (*n* = 348)	Rural (*n* = 1,386)
Variable	%	%
Father with less than 6 years of formal education	8.6	37.6
Mother with less than 6 years of formal education	13.0	43.0
Stunted growth	9.2	29.7
Wealth index under 0.5	4.9	54.7

There was very little attrition from the sample. At 5 years, 98.8% of the 2,000 children who were recruited as infants were retained. At 15 years, 96.8% of the initial sample, and 98.1% of the sample at 5 years, were retained. However, data for outcomes at 15 years of age should be interpreted in the knowledge that 20.1% of children had dropped out of school by the time these were collected.

When the age 5 data were collected, 77.7% of the sample was beyond preschool age and had recently started primary school. The vast majority of children had attended a preschool (Table 3), and the vast majority of these had attended a public preschool. The average duration of attendance was one-and-a-half years.

**Table 3:** Vietnamese children’s preschool attendance between 3 and 5 years of age.

Preschool education	Mean	(SD)	%
Did not attend preschool			8.4
Attended preschool			91.6
Age when first attended preschool (months)	44.6	(9.2)	
Hours per week	32.4	(15.2)	
Months attended	18.9	(9.7)	
Dose (hours)	2754.5	(2110.1)	
Public preschool			86.0
Private (for profit) preschool			10.5
Private (Not for profit) preschool			1.0
Informal community and other preschool			2.3

### 2.4. Measures

#### 2.4.1. Preschool attendance and dose

At 5 years of age, primary caregivers answered single items that asked whether, since the age of 3 years, their child had ever attended preschool; the age in months at which they started attending preschool; the age in months at which they stopped attending preschool; the number of hours their child attended per week; the type of preschool they attended (e.g., public, private); whether the child had begun attending school; and the age at which they commenced school. An approximate measure of dose was calculated as the product of the derived number of months the child attended preschool, the reported hours of attendance per week, and 4 weeks/month. Parents were also asked for the reasons why their child did or did not attend preschool.

#### 2.4.2. Outcome variables

The timing of data collection for outcome variables is summarized in Table 4.

**Table 4:** Timing of outcome measures.

	Child age in four rounds of data collection			
Outcome	5 years	8 years	12 years	15 years
Cognitive				
Receptive vocabulary	x	x	x	x
Basic numeracy skills	x			
Mathematics tests		x	x	x
Socio-emotional				
Life satisfaction	x	x	x	x
Self-efficacy			x	x
Self-esteem			x	x
Peer relations			x	x
Parental relations			x	x

##### 2.4.2.1. Receptive vocabulary

A Vietnamese language adaptation (Cueto and León, 2012) of the *Peabody Picture Vocabulary Test-III* (PPVT-III) (Dunn and Dunn, 1997) was administered at 5 and 8 years of age. Although the adaptation showed good psychometric properties during pilot testing (Cueto and León, 2012), a subsequent analysis when the children were 8 years old identified poorly performing items and issues concerning the sequence of words. This led to the decision to correct scores from 5 and 8 years for the poorly performing items, and modify assessments at later ages to shorten the number of items and change their order to better reflect item difficulty in Vietnamese. The resulting test, which was used at 12 and 15 years, could no longer be described as an adaptation of the PPVT-III but remained a test of receptive vocabulary (Leon and Singh, 2017). In all cases, the child’s task was to indicate which of four drawings depicted the word spoken by the administrator. The tests comprised 17 sets of 12 words of increasing difficulty. Rasch scores were used at 5 and 8 years of age; percentage correct scores were used at 12 and 15 years.

##### 2.4.2.2. Numeracy and mathematical skills

At 5 years, basic numeracy skills were assessed by a 14-item version of the quantity sub-test from the *Cognitive Development Assessment* (CDA-Q) (Cueto et al., 2009). This excluded CDA-Q Question 6 due to its poor measurement fit. Children selected the most appropriate image for a concept (e.g., a few, most, nothing, equal, and a pair) from three or four alternatives (e.g., “Point to the plate that has a few cupcakes”). Rasch scores were created from the sum of correct answers.

At 8, 12, and 15 years of age, custom designed mathematics tests were administered. Items were drawn from the *Program for International Student Assessment* (PISA) and the *Trends in International Mathematics and Science Study* (TIMMS) (Dawes, 2020). These tests are used internationally to track children’s mathematical ability (Cueto and León, 2012). Test items increased in difficulty at each age point.

Revollo and Scott (2022) provide details about the pilot testing of each of these measures in Vietnam. Additional details about the vocabulary and numeracy/mathematics measures are provided by Dawes (2020).

##### 2.4.2.3. Life satisfaction

*Cantril’s Ladder* (Cantril, 1965) is a single-item measure of the child’s current global life satisfaction: “Suppose there are nine steps in this ladder, in which the top step of the ladder (score of 9) represents the best possible life, and the bottom step (score of 1) represents the worst possible life… Where on the ladder do you feel you personally stand at the present time?”

##### 2.4.2.4. Self-concept and relationships

Self-efficacy was measured using an adaptation of the *General Self-efficacy Scale* (Schwarzer and Jerusalem, 1995) which contains 10 items designed for use with adolescents and adults (e.g., “It is easy for me to stick to my aims and accomplish my goals”). Self-esteem was measured using a subset of eight items from the *Self-Description Questionnaire II* (*SDQII*; Marsh, 1990a; e.g., “A lot of things about me are good”). Peer relations were measured using a subset of eight items from the *Self-Description Questionnaire I* (*SDQI*; Marsh, 1990b; e.g., “I am popular with kids of my own age”). Parent relations were measured using a subset of eight items from the *Self-Description Questionnaire II* (*SDQII*; Marsh, 1990a; e.g., “I get along well with my parents”). All items on the four scales assessing self-concept and relationships were rated on 4-point scales (strongly disagree to strongly agree). Previous research has reported that these scales have adequate factor structure in Vietnam (Scholz et al., 2002; Yorke and Ogando Portela, 2018). In the current study, these tests showed adequate internal consistency (Table 5). Yorke and Ogando Portela (2018) provide details about the pilot testing of each of these measures in Vietnam.

**Table 5:** Internal consistency of the self-concept and relationship measures when Vietnamese children were 12 and 15 years of age.

Scale	Cronbach alpha	
	12 years	15 years
Self-concept
Self-efficacy (10 items)	0.70	0.71
Self-esteem (8 items)	0.70	0.71
Relationships
Peer relations (8 items)	0.72	0.70
Parent relations (8 items)	0.81	0.84

## 3. Results

### 3.1. Analysis plan

Different statistical analyses were relevant to the four analytic approaches adopted in this study. Research Questions 1 and 2 were addressed by comparing outcomes for children who did and did not attend preschool using analysis of covariance to statistically control for differences between the groups in age, gender, urban/rural location and family wealth. Research Questions 2 and 3, were addressed by hierarchical regression analyses that included only those children who had attended preschool. Child age was entered in Step 1. Step 2 added the dose of education received by children (continuous data in hours). Statistically significant correlations were judged to provide evidence of a meaningful dose-dependent relationship between preschool education and child outcomes if they accounted for more than 2% of variance. The third and fourth analytical approaches involved planned analyses that addressed Research Questions 5 and 6. Prior to these analyses, preschool dose was classified into three groups: low (1 to 999 h, equivalent to half-time enrolment for up to 1 year), intermediate (1,000 to 2,999 h, equivalent to half-time enrolment for up to 1 year at 3 years of age and full-time enrolment for up to 1 year at 4 years of age) and high (3,000 h or more). All planned comparisons covaried for differences between the groups in child age.

The large sample size resulted in very high statistical power with the potential to identify trivial effects. Because of this, and the large number of comparisons needed to answer the research questions, *p* < 0.01 was adopted as the criterion for statistical significance. Analyses were conducted using SPSS 28.0.1.1. Effect sizes are reported as partial eta squared (*η_p_*^2^) or *R*^2^.

### 3.2. Children who did and did not attended preschool

**Figure 1:**
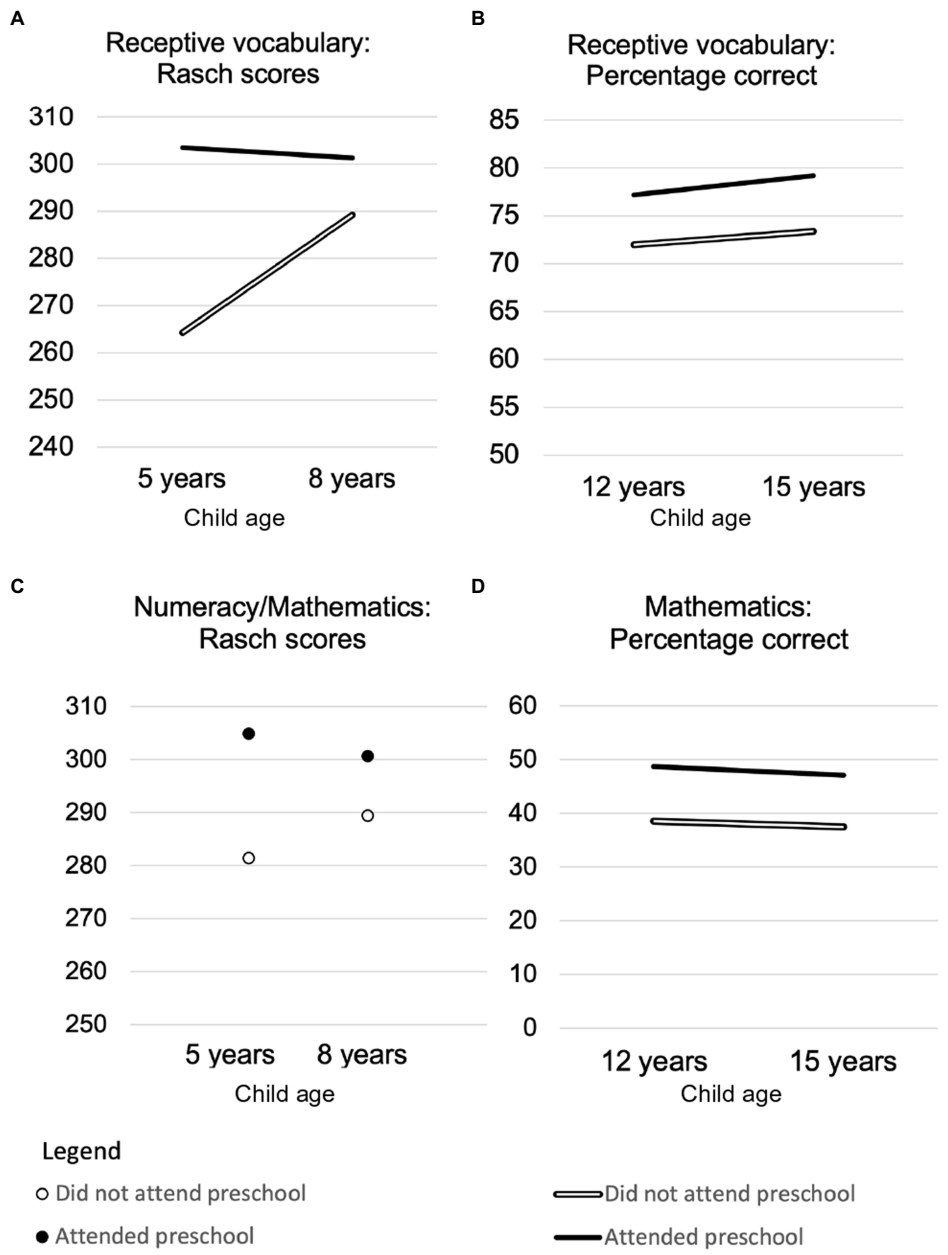
Longitudinal changes in cognitive outcomes at four ages for Vietnamese children who did (*n* = 164) and did not attend preschool (*n* = 1,562).

**Figure 2:**
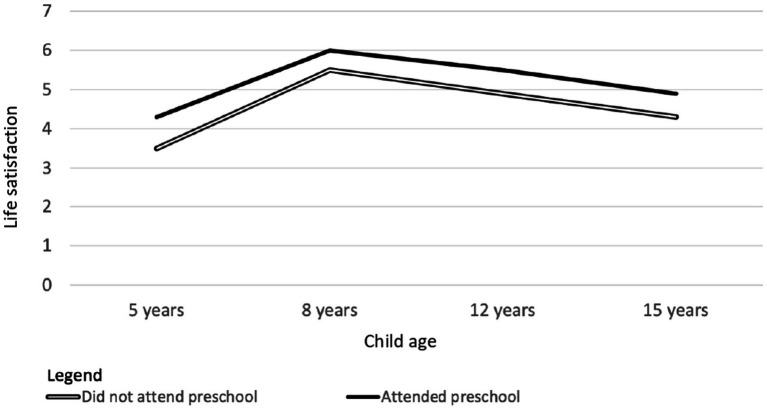
Ratings of life satisfaction at 5, 8, 12, and 15 years of age by children who did (*n* = 1,562) and did not attend preschool (*n* = 163).

Children who did and did not attend preschool differed in several demographic variables (Table 6). Therefore, differences between the groups in the four demographic variables (child age, gender, urban/rural location, and family wealth) that were most strongly related to outcomes were statistically controlled in the analyses. Despite this, children who attended preschool (*n* = 1,562) had larger vocabularies at 5, 8, 12, and 15 years (Figure 1, Panels A and B), greater mathematics skills at 5, 8, and 12 years (Figure 1, Panels C and D), and higher life satisfaction at 5 and 12 years of age (Figure 2) than the small number of children who did not attend preschool (*n* = 164). Statistics are summarized in Supplementary Table 1. Although effect sizes were small in all cases, many were likely to be meaningful. For example, even though the mathematics skills assessed during adolescence were not taught until many years after children began formal schooling, at both 12 and 15 years of age, the scores on mathematics tests for children who did not attend preschool were about 10 percentage points lower than those for children who attended preschool. There were no difference between the groups in self-esteem, or the quality of relationships with peers or parents at either of the ages in which these were assessed (12 and 15 years). However, at 15 years of age, children who attended preschool had higher self-efficacy than those who did not. Again, the effect size was very small.

**Table 6:** Demographic characteristics of Vietnamese children who did and did not attend preschool.

	Did not attend preschool (*n* = 164)			Attended preschool (*n* = 1,565)		
Variable	%	M	(SD)	%	M	(SD)
Child age (months)		60.4	(3.9)		63.9	(3.6)
Female	47.6			44.0		
Rural place of residence	92.7			70.3		
Family wealth index		0.336	(0.216)		0.517	(0.194)
Father with less than 6 years of formal education	60.6			28.7		
Mother with less than 6 years of formal education	68.9			33.7		

In answer to Research Question 1, children who attended preschool had a small advantage in vocabulary, numeracy and life-satisfaction at 5 years of age. In answer to Research Question 2, all of these advantages persisted at most later ages. Indeed, the advantage in receptive vocabulary associated with preschool attendance persisted at all later ages.

#### 3.2.1. Reasons why children did and did not attend preschool

In a context in which the vast majority of children attend preschool, parents who choose not to send their child to preschool may have distinctive personal beliefs that are not captured by the demographic characteristics used as covariates in the analyses above. Parents were asked why they had chosen to send or not send their child to preschool (Table 7). Because many parents offered more than one reason, responses sum to more than 100%.

**Table 7:** Vietnamese parents’ self-reported reasons for choosing to send, or not send, their child to preschool.

Reasons why parents chose to send their child to preschool	%	Reasons why parents chose not to send their child to preschool	%
Education		Child-minding	
To help the child prepare for school	86.3	Child was cared for by others	65.6
To receive a good education	29.0	Financial	
Social		Could not afford fees/ transport	46.8
It is important for the child to mix with other children of the same age	45.7	Child motivation	
Child motivation		The child did not want to go	45.1
Child wanted to go	9.5	Other	
Child-minding		The child was too young	43.0
To provide safe care while parents worked for money	22.5	It was not necessary	18.2
To keep the child out of the way at home	12.9	There was no preschool within easy reach	17.0
No relatives or friends available to help look after the child	2.1		
To provide safe care while parents care for others	0.5		

Most parents who sent their child to preschool did so to support their child’s education. Six reasons accounted for almost all the explanations offered by parents whose child did not attend preschool. The most common reason was that they did not require the childcare that preschools provide. Indeed, many parents who sent their child to preschool provided explanations suggesting that they too perceived that preschools provide child-minding rather than education.

The prominence of financial explanations for not sending a child to preschool is consistent with these families’ lower median wealth index score at 5 years of age (Table 6). However, full and partial subsidies for preschool fees are available for socio-economically disadvantaged families. For 6% of families whose children attended preschool, these completely covered the cost of fees. In addition, many families (19.1%) with wealth index scores lower than the median for non-attenders nevertheless sent their children to preschool; and many families (19.1%) with wealth index scores above the median for children who attended preschool did not send their children to preschool. No parent indicated that a mismatch between home and preschool in culture or language, or concerns about the content of the preschool curriculum or quality of instruction influenced their decision.

One explanation for not sending a child to preschool is difficult to interpret. About 1 in 5 of the parents who did not send their child to school indicated that their child was too young have attended preschool when they were between 3 and 5 years of age, even though these are the ages for which preschool education is offered in Vietnam (Thang and Hang, 2018).

### 3.3. Association between dose of preschool education and outcome variables

#### 3.3.1. Revised analysis plan

Decisions concerning the inclusion of confounding variables in the regression analyses were complicated by the moderate to strong associations between urban–rural location, family wealth index, and mothers’ and fathers’ levels of education. To avoid introducing collinearity into the analyses, only the strongest predictor of outcomes among these variables, family wealth index, was included.

Although the Young Lives Study did not recruit participants from communes in the highest wealth quintile, some individual families had high wealth index scores. Indeed, family wealth scores when the children were 5 years of age (range: 0.006–0.935) covered almost all the possible range (0–1.0). Preliminary examination of the data revealed that the relationship between preschool dose and outcomes differed for children from families with lower and higher wealth index scores, and that it was more informative to display the difference in these patterns than to report the B and ß values that regression analyses yielded for family wealth. Therefore, a median split was made in the sample to form lower family wealth (0–0.526) and higher family wealth groups (0.527–1.0). The planned hierarchical linear regression analyses exploring relationships between the dose of preschool education children received and the outcome variables were conducted separately for children in the two wealth groups. Child age at the time of the relevant data collection point was entered in Step 1 of the regression. Dose of preschool education (hours as a continuous variable) was entered in Step 2.

#### 3.3.2. Association between preschool dose and outcome variables

The strength of associations between preschool dose and outcome variables is summarized in Table 8. Additional details concerning the regression analyses can be found in Supplementary Table 2.

**Table 8:** Associations between the dose of preschool education received by Vietnamese children from poorer and wealthier^ families and their cognitive and socio-emotional outcomes at four ages after controlling for individual differences in child age.

Outcome	*R*^2^ change							
	Lower family wealth (*n* = 864)				Higher family wealth (*n* = 870)			
	Child age (years)				Child age (years)			
	5	8	12	15	5	8	12	15
Cognitive								
Receptive vocabulary	0.042**	0.077**	0.032**	0.041**	0.161**	0.033**	0.029**	0.009*
Quantity/Mathematics	0.012**	0.001	0.011*	0.003	0.039**	0.040**	0.037**	0.075**
Socio-emotional								
Life satisfaction	0.029**	0.008	0.028**	0.007	0.002	0.001	<0.001	<0.001
Self-efficacy	–	–	0.004	0.005	–	–	0.001	0.020**
Self-esteem	–	–	0.005	<0.001	–	–	<0.001	0.018**
Relationship with peers	–	–	<0.001	<0.001	–	–	0.003	0.016**
Relationship with parents	–	–	<0.001	0.013*	–	–	0.003	0.013*

–, outcome was not assessed at this age.

^ The Wealth Index was calculated from information concerning quality of housing, access to basic services and ownership of consumer durables (Young Lives, 2002). Scores range between 0 and 1, with higher scores indicating higher wealth. Lower family wealth: wealth index scores between 0 and 0.526; Higher family wealth: wealth index scores between.527 and 1.0.

* *p* < 0.01.

** *p* < 0.001.

For children from families with lower wealth index scores, preschool education showed a small but meaningful dose-dependent association with receptive vocabulary at all four ages, and with life satisfaction at 5 and 12 years. However, preschool dose failed to account for more than 2% of the variance in scores for numeracy/mathematics skills, self-efficacy, self-esteem, peer relations or parent relations at any age, even though a small number of R^2^ change values met the criterion for statistical significance.

For children from families with higher wealth index scores, preschool education showed a meaningful dose-dependent association with both receptive vocabulary and numeracy/mathematics skills at all four ages. However, preschool dose failed to account for more than 2% or more of the variance in scores for life satisfaction, self-efficacy, self-esteem, peer relations or parent relations at any age, even though a small number of R^2^ change values met the criterion for statistical significance.

Although most effect sizes in both groups were small, several were likely to be of practical significance. Preschool dose accounted for more than 16% of the variance in receptive vocabulary scores when children from wealthier families were 5 years of age and 7.5% of the variance in their mathematics score when they were 15 years of age. Preschool dose also accounted for slightly more than 7.5% of the variance in the vocabulary scores of when children from poorer families were 8 years of age.

In answer to Research Question 3, at 5 years of age there were meaningful associations of small or medium magnitude between the dose of preschool education that children received and their receptive vocabulary (children from both poorer and richer families), basic numeracy skills (children from richer families only) and life-satisfaction (children from poorer families only). In answer to Research Question 4, each of these dose-dependent associations were also observed at two or more subsequent ages.

#### 3.3.3. Longitudinal changes in the association between preschool dose and outcome variables

**Figure 3:**
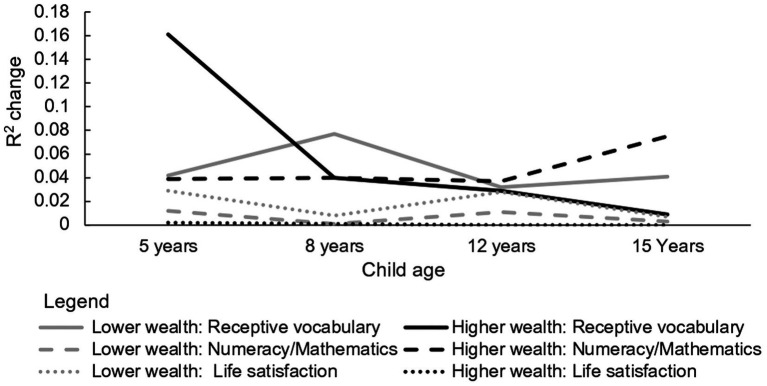
Longitudinal changes in the strength of association between the dose of preschool education received by children from families with lower and higher wealth and their receptive vocabulary, numeracy/mathematics and life satisfaction scores after controlling for individual differences in child age.

Changes in the strength of the association between preschool dose and receptive vocabulary, numeracy/mathematics skills and life satisfaction are plotted in Figure 3. These too show differences between children from families with lower and higher wealth index scores. Only one of the longitudinal patterns is consistent with a fading of the benefits of preschool education over time: the decrease in the strength of the association between preschool dose and receptive vocabulary scores for children from wealthier families. Indeed, the strength of the association with preschool dose peaked many years after children began school for two outcomes: life satisfaction ratings by children from poorer families and numeracy/mathematics skills of children from wealthier families.

#### 3.3.4. Children not enrolled in school at 15 years of age

A separate analysis of the dose-dependent relationship between preschool education and outcomes at 15 years of age was conducted for children who were no longer enrolled in school at this age. These children were more likely to be male, live in rural areas, be from families with a lower wealth index score, and be slightly older than their peers who continued their schooling (Supplementary Table 3). The small sample size precluded separate analyses for out-of-school children with higher and lower wealth index scores. Therefore, child age and family wealth were entered in Step 1 of the hierarchical linear regression. When dose of preschool education (continuous data in hours) was entered in Step 2, only one meaningful dose-dependent relationship was found. The dose of preschool education accounted for 4.3% of the variance in the quality of out-of-school children’s relationship with their parents (Supplementary Table 4).

### 3.4. Ameliorating rural disadvantage

The original plan was to compare the outcomes of urban and rural children within each of three preschool dose categories: low (1 to 999 h), intermediate (1,000 to 2,999 h), and high (3,000 h or more). However, because the size of the urban sample was small and almost all urban children received a high dose of preschool education, the urban low and intermediate preschool dose groups had such small sample sizes that they could not yield meaningful data. Therefore, comparisons between rural and urban children who received similar doses of preschool education could be conducted only for children in the high dose group.

#### 3.4.1. Do rural children who receive a high dose of preschool education continue to be developmentally disadvantaged compared to their urban peers?

**Figure 4:**
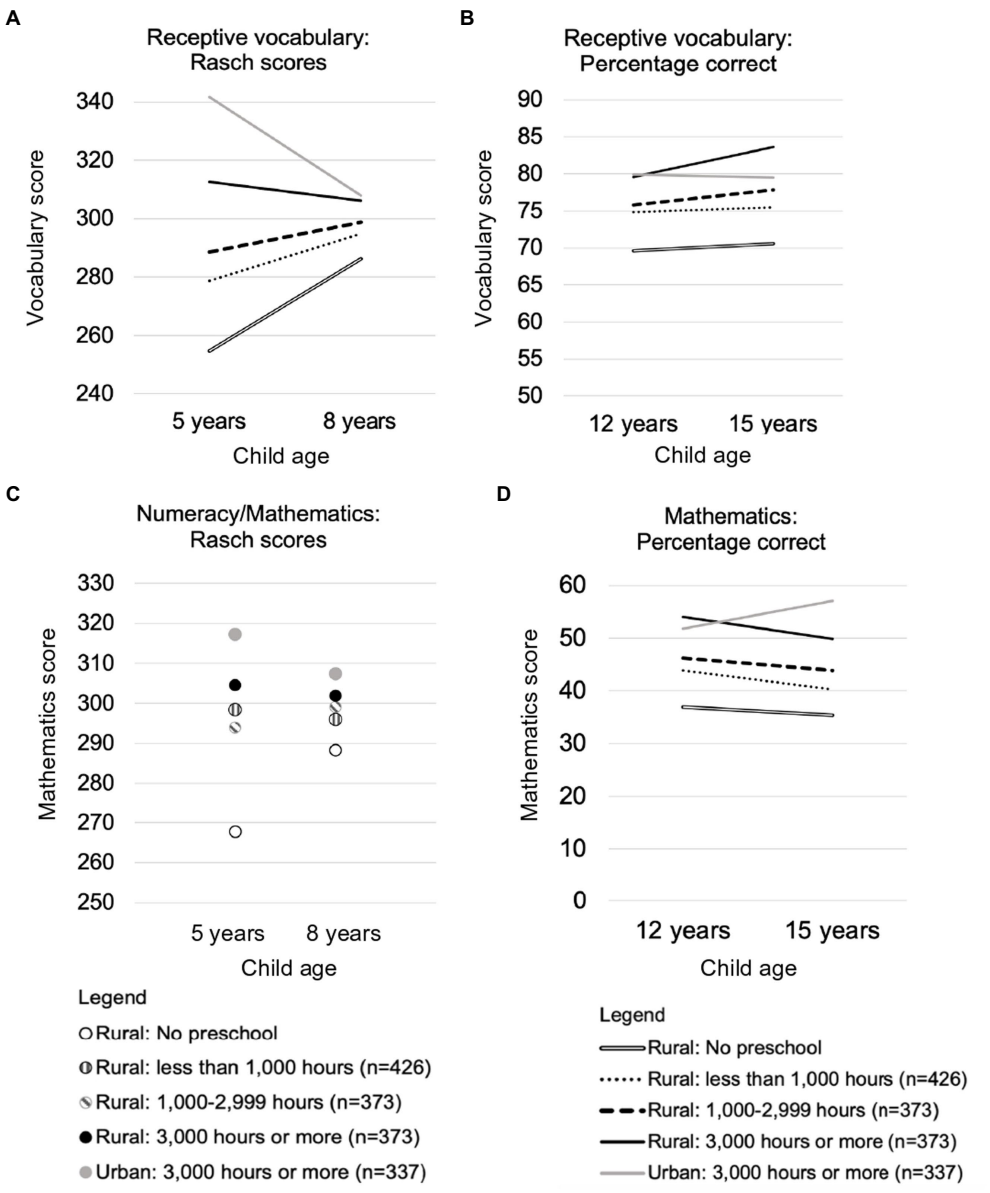
Longitudinal changes in cognitive outcomes for rural children who received different doses of preschool education and urban children who received a high dose of preschool education.

Outcomes for urban (*n* = 337) and rural children (*n* = 373) who received a high dose of preschool education were compared after statistically controlling for differences in child age between the two groups. Urban children showed an advantage in basic numeracy and receptive vocabulary scores at 5 years of age, and in mathematics scores at 8 and 15 years of age (Figure 4 and Table 9). There was no difference between urban and rural children in receptive vocabulary scores at 8 and 12 years of age, or in mathematics scores at 12 years of age. Rural children had higher life satisfaction ratings at 5 years, and higher receptive vocabulary scores and self-esteem at 15 years of age. Descriptive statistics are summarized in Supplementary Table 5. Urban children’s advantage in receptive vocabulary at 5 years of age and in mathematics at 8 years of age had medium effect sizes. In all other cases, the effect size was small. There were no differences between the groups in self-efficacy, peer relations or parent relations at 12 or 15 years of age, and no difference in self-esteem at 12 years of age (Supplementary Table 5).

**Table 9:** Summary of analyses comparing outcomes between rural (*n* = 373) and urban (*n* = 337) Vietnamese children who received a high dose of preschool education.

Child age and outcome	Statistic	Sig.	*η_p_* ^2^
5 years
Receptive vocabulary: Rasch scores	*F*_(1,575)_ = 74.2	**	0.114
Basic numeracy: Rasch scores	*F*_(1,628)_ = 34.2	**	0.052
Life satisfaction	*F*_(1,654)_ = 6.8	**	0.010
8 years
Receptive vocabulary: Rasch scores	*F*_(1,602)_ = 5.3	ns	
Mathematics: Rasch scores	*F*_(1,640)_ = 57.2	**	0.082
Life satisfaction	*F*_(1,661)_ = 3.6	ns	
12 years
Receptive vocabulary: percentage correct	*F*_(1,610)_ = 0.2	ns	
Mathematics: percentage correct	*F*_(1,602)_ = 0.7	ns	
Life satisfaction	*F*_(1,661)_ = 3.6	ns	
Self-efficacy	*F*_(1,608)_ = 0.5	ns	
Self-esteem	*F*_(1,607)_ = 5.5	ns	
Peer relations	*F*_(1,609)_ = 1.4	ns	
Parent relations	*F*_(1,600)_ = 1.2	ns	
15 years
Receptive vocabulary: percentage correct	*F*_(1,634)_ = 25.3	**	0.038
Mathematics: percentage correct	*F*_(1,629)_ = 20.3	**	0.031
Life satisfaction	*F*_(1,632)_ = 5.1	ns	
Self-efficacy	*F*_(1,623)_ = 0.1	ns	
Self-esteem	*F*_(1,617)_ = 13.2	**	0.021
Peer relations	*F*_(1,617)_ = 3.1	ns	
Parent relations	*F*_(1,618)_ < 0.1	ns	

** *p* < 0.001.

ns: not significant, *p* ≥ 0.01.

In answer to Research Question 5, even when rural children received high doses of preschool education, they continued to have lower scores for receptive vocabulary and numeracy/mathematics than their urban peers at the age for school entry. These disadvantages did not always persist. In particular, by age 8, the two groups differed only in mathematics skills, and by 12 years of age there was no difference between the two groups on any measure (although some differences re-emerged at 15 years of age). It was also notable that when differences were found, rural children were not always at a disadvantage (Figure 4, Panel B).

#### 3.4.2. Do rural children benefit from a low dose of preschool education?

**Figure 5:**
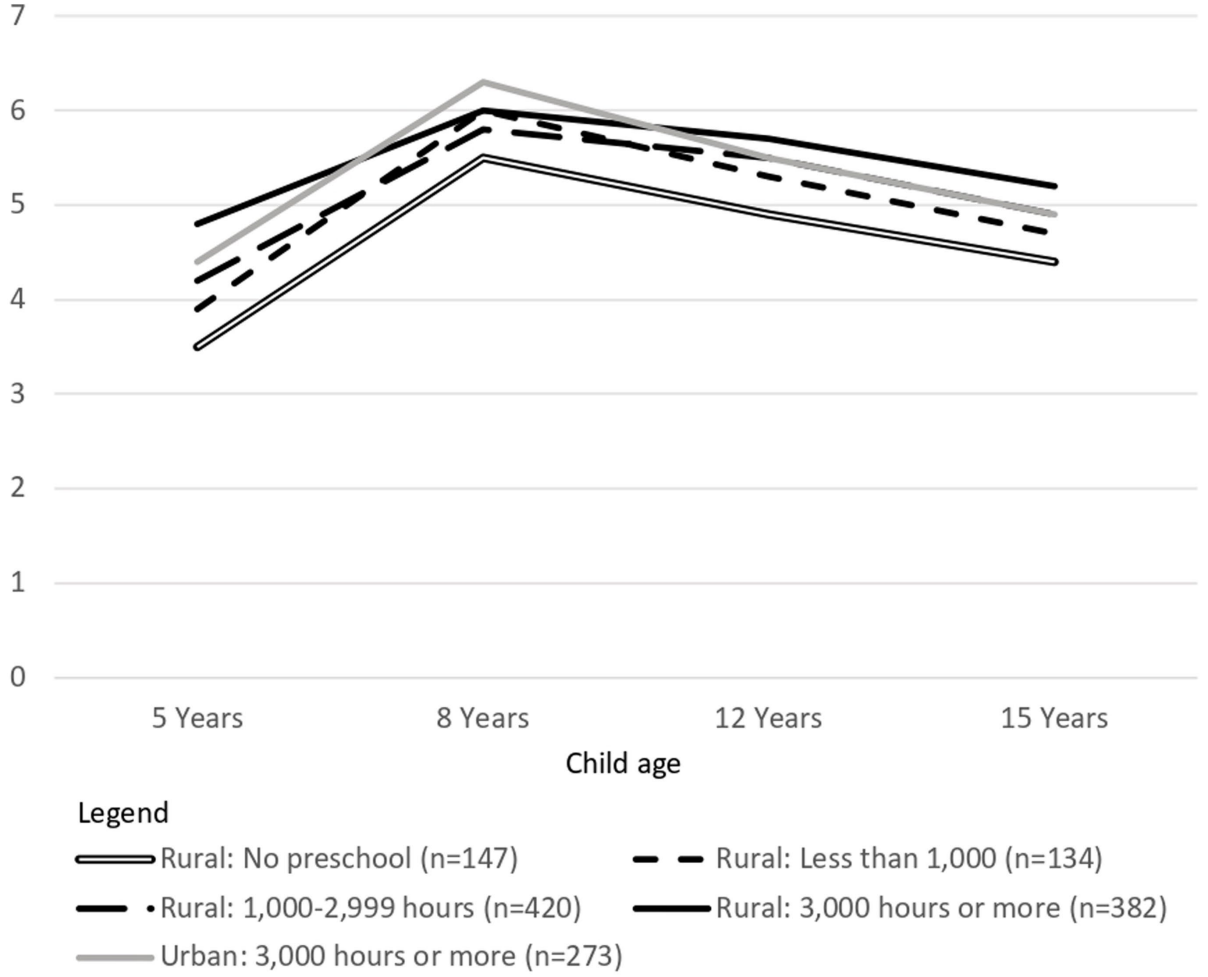
Life satisfaction at four ages for rural children who received different doses of preschool education and urban children who received a high dose of preschool education.

Because a low dose of preschool education may be all that is feasible and sustainable in many low- and middle-income countries, the final analyses compared outcomes for rural children who did not attend preschool (*n* = 152) with those of rural children who received a low dose (*n* = 426). Age and wealth differences between the two groups were statistically controlled. Rural children who received a low dose of preschool education had higher scores than their peers who did not attend preschool on measures of receptive vocabulary at 5, 8, and 15 years of age and higher scores for basic numeracy skills and mathematics tests at 5, 8, and 12 years (Figure 4 and Table 10). Descriptive statistics are provided in Supplementary Table 5. In all cases, the effect size was small. Despite the apparent trend in Figure 5, there were no group differences in life satisfaction scores. It was obvious from visual inspection that there were also no differences between these groups in the self-concept or relationship measures (Supplementary Table 5).

**Table 10:** Summary of analyses comparing outcomes between rural Vietnamese children who received a low dose of preschool education (*n* = 426) and those who did not attend preschool (*n* = 152).

Child age and outcome	Statistic	Sig.	*η_p_* ^2^
5 years
Receptive vocabulary: Rasch scores	*F*_(1,403)_ = 8.4	*	0.020
Basic numeracy: Rasch scores	*F*_(1,522)_ = 12.5	**	0.023
Life satisfaction	*F*_(1,579)_ = 1.5	ns	
8 years
Receptive vocabulary: Rasch scores	*F*_(1,530)_ = 26.3	**	0.047
Mathematics: Rasch scores	*F*_(1,531)_ = 24.1	**	0.043
Life satisfaction	*F*_(1,564)_ < 0.1	ns	
12 years
Receptive vocabulary: percentage correct	*F*_(1,558)_ = 4.4	ns	
Mathematics: percentage correct	*F*_(1,531)_ = 7.9	*	0.015
Life satisfaction	*F*_(1,565)_ = 4.4	ns	
15 years
Receptive vocabulary: percentage correct	*F*_(1,565)_ = 7.7	*	0.013
Mathematics: percentage correct	*F*_(1,535)_ = 1.4	ns	
Life satisfaction	*F*_(1,568)_ = 1.9	ns	

* *p* < 0.01.

** *p* < 0.001.

ns: not significant, *p* ≥ 0.01.

In answer to Research Question 6, rural children who received a low dose of preschool education showed a small but long-lasting advantage in receptive vocabulary and numeracy/mathematics skills compared to their peers who did not attend preschool even when wealth differences between these groups were accounted for.

## 4. Discussion

The current study used four analytic approaches to examine associations between preschool education delivered at scale under the resource constraints that apply in low- and middle-income countries and diverse outcomes for children. It did this in a context in which there were national curricula and teaching standards for preschools and schools, very high rates of preschool attendance, and subsidies for preschool and school fees for economically disadvantaged children. These conditions and the use of longitudinal data limited the heterogeneity in children’s experiences that can obscure patterns in outcomes.

### 4.1. Benefits associated with preschool attendance

Findings from each of the four analytic approaches supported the conclusion that preschool education is associated with both short- and long-term benefits for children’s cognitive development. First, children who did not attend preschool showed little evidence of catch-up to children who had attended preschool, even after differences between these groups in age, gender, urban–rural location and family wealth were accounted for. There was no catch-up in receptive vocabulary at any age, and catch-up in mathematics at only one age. Second, for both children from poorer and wealthier families, there was a small to moderate positive association between dose of preschool education and receptive vocabulary at every age. For children from wealthier families, there was also a positive association between preschool dose and numeracy/mathematics skills at every age. Third, high doses of preschool education ameliorated some domains of developmental disadvantage for rural children, when these were compared with urban children with similarly high doses of preschool education. Although these rural children showed no immediate “catch-up,” the size of their vocabularies was comparable to or greater than those of their urban peers at all later ages, and their mathematics skills were comparable at one later age. It may not be feasible or sustainable for many low- and middle-income countries to deliver the high doses of preschool education observed in this research. Therefore, the results of the final analysis were encouraging. These showed that even when rural children received only a low dose of preschool education, their scores on most cognitive outcomes at most ages were higher than those of their peers who did not attend preschool. The four analytic approaches produced less consistent findings about the association between preschool education and children’s life satisfaction. However, several analyses showed positive associations at one or more ages.

Although the magnitude of most effects identified in the four analytic approaches was small, many were likely to be meaningful at both an individual and national level. For example, despite the many family, child, and school factors that contribute to cognitive development, individual differences in the dose of preschool education received by children from wealthier families accounted for more than 16 percent of the variance in their receptive vocabulary scores at 5 years of age, and 7.5% of the variance in their mathematics scores at 15 years of age. It is notable that several benefits associated with preschool attendance persisted for at least 10 years. Indeed several of the strongest associations reflected delayed effects that would not have been identified in shorter-term longitudinal research. The longevity and magnitude of the effects observed in this research equaled or surpassed those achieved by many better-resourced generic preschool education programs in high-income countries (e.g., Li et al., 2020; Burchinal et al., 2022).

### 4.2. Differences across outcomes

As has been reported in previous research (e.g., Barnett, 1998; Sylva et al., 2004) different outcomes showed different patterns of association with preschool education. Across the four analytic approaches, there was a tendency for preschool education to be more strongly associated with receptive vocabulary than with mathematics. This may be an artifact of the different emphasis these skills receive in the Vietnamese preschool curriculum. Language and social and emotional development are two “pillars” of the national preschool curriculum, but numeracy skills are only one sub-target within the “pillar” of cognitive development (Ministry of Training and Education, Vietnam, 2009).

The current findings concerning developmental changes in the association between preschool education and children’s outcomes are also noteworthy. First, they are consistent with previous research indicating that preschool education shows outcome-specific patterns of persistence and fading (e.g., Apps et al., 2013; Li et al., 2020). For example, the dose-dependent association between preschool education and mathematics was more stable than that for vocabulary size. Second, even though the strength of some associations faded, the timing of the greatest benefit may have important implications for later development. For example, the strongest association between preschool dose and receptive vocabulary was found at 5 years of age. Previous research has reported that individual differences in the pace of vocabulary development during the preschool years predict differences in the thickness of some areas of the cerebral cortex when children are 8 and 10 years of age (Asaridou et al., 2017).

Both social justice and economic rationales underlie investments in preschool education. If low levels of school readiness among children from disadvantaged communities can be overcome, this may begin a virtuous cycle in their development. An increased ability to benefit from schooling may lead to higher academic attainment, which may open greater employment opportunities, and break the intergenerational transmission of poverty (e.g., Conti and Heckman, 2014). If these effects can be achieved at scale, they can make a significant contribution to the economic development and stability of nations (World Bank, UNESCO, and UNICEF, 2021). The current findings suggest that even under resource-constrained conditions, preschool education may achieve some social justice goals. High doses of preschool education were associated with a more “level playing field” for children from disadvantaged rural communities. For at least the following 10 years, they achieved outcomes in some domains of development that were comparable to, or better than, those achieved by their peers living in more advantaged urban communities, even when differences between these groups in wealth and parental education were not statistically controlled. Moreover, children from disadvantaged communities made gains in receptive vocabulary and mathematics skills when they received only a low dose of preschool education, and these gains too were detectable for the following 10 years. For example, at both 12 and 15 years of age, rural children who received a low dose of preschool education achieved scores for both receptive vocabulary and mathematics that were about 5 percentage points higher than those for rural children who had not attended preschool. Despite these findings, it needs to be acknowledged that preschool attendance may not always be associated with greater equity in children’s later educational outcomes. In the current study, the strength of associations between preschool dose and numeracy/mathematics scores for children from poorer families were systematically smaller than those for children from wealthier families. Nevertheless, if the Young Lives Study continues, it may be possible to determine whether the benefits associated with generic preschool education allow children to achieve livelihoods that generate greater national wealth and prevent the intergenerational transmission of poverty.

### 4.3. Cultural and contextual issues in measurement

Accumulated evidence raises questions about the utility of the measures of self-efficacy, self-esteem, and the quality of children’s relationships with peers and parents. Children’s scores showed little variability across these measures, or across locations, doses of preschool education or other variables. This apparent lack of sensitivity may reflect the challenge of selecting measures that are culturally and contextually appropriate across countries. All four measures were designed for use in the developmental contexts encountered by children in high-income countries and in cultures that value individualism. In contrast, Vietnamese culture is influenced by its Confucian heritage, endorses collectivist values, and cultivates modesty and humility in children (e.g., Binh, 1975). Consequently, it may be culturally inappropriate for Vietnamese children to make the claims contained in items such as “If someone opposes me, I can find the means and ways to get what I want” (self-efficacy), “Overall, I have a lot to be proud of” (self-esteem) and “I have more friends than most other kids” (peer relations). Responses to other items are likely to have been influenced by context. For example, the item “My parents and I spend a lot of time together” (parent relations) may reflect necessity rather than a preference in a context in which 44% of 15-year-old children lived in dwellings containing only one or two rooms. In summary, reflection on the self-concept and relationship measures has raised questions about the validity of some items when they are applied in Vietnamese contexts. These questions suggest that caution should be exercised when drawing conclusions from the current research about the association between preschool education and socio-emotional development. It is also possible that preschool education in Vietnam has its strongest associations with domains of socio-emotional development, such as filial piety (Nguyen, 2022), that are particularly valued in Vietnamese culture but were not assessed in the current study.

### 4.4. Limitations of the current study

Several limitations of the current research need to be considered when interpreting its findings. First, despite the large sample size, there were too few participants with specific characteristics to allow some important questions to be answered. It was not possible to derive meaningful data for urban children or children from ethnic minority groups who did not attend preschool or who received low or intermediate doses of preschool education. Second, in the current sample, dose of preschool education was confounded with preschool starting age. Very few children who began preschool education withdrew before the age for school entry, and there was limited variability in the number of hours of attendance per week among children who began preschool at 3 years of age. Consequently, the current analyses cannot inform the debate about the optimal age for children to begin preschool education. Nguyen and Goutte (2022) analysis of the same dataset focused on this question. Third, despite the Vietnamese government’s attempts to standardize preschool education, it is likely that there was variability in its quality. This could not be accounted for in the current analyses since the *Young Lives Study* did not collect objective data on preschool quality. A single item asked parents to rate the “standard of care and teaching” provided by their child’s preschool. It is difficult to interpret the data this item generated for three reasons: the item is “double-barreled”; two-thirds of the sample assigned the same rating (“Good”); and the basis on which the 11 percent of mothers who had not attended either a preschool or a school made judgments on the quality of teaching is unclear. Finally, the results provide limited insights for Vietnamese policymakers. The *Young Lives* sample is not nationally representative, and several important educational reforms have taken place since the children in its sample attended preschool. For example, preschool teachers now require a university-level teaching qualification; there is more effective implementation of child-centered pedagogies; and the national government adopted an ambitious plan to further promote the development of early childhood education between 2018 and 2025 (Phan, 2012; Prime Minister, Vietnam, 2018).

### 4.5. Policy implications

Despite this, the current findings have a number of policy implications for other low- and middle-income countries. First, they suggest that universal preschool programs can be a good short- and longer-term investment even under resource-constrained conditions. However, policymakers in these countries need to make difficult decisions about the most efficient use of the limited funding available to support child development. Vietnam did not achieve universal preschool education without making significant sacrifices in other areas. In 2008, in the lead-up to making preschool education mandatory, Vietnam dedicated 18% of its national budget to education, including preschool education. This commitment remained between 17 and 19 percent in each of the 5 years following the implementation of this policy (UNESCO Institute for Statistics, 2022a). This is a higher percentage than was spent during the same period by many high-income countries including Australia, Canada, Finland, and Germany (UNESCO Institute for Statistics, 2022b), which were not also attempting to address critical shortages in basic health services, nutrition, and public infrastructure. To make evidence-informed budgetary decisions, policy makers would need to have access to comparative analyses of the return on investments from preschool education, child nutrition, improvements in the quality of basic education, and other services and infrastructure. For example, in the case of Vietnam, where chronic malnutrition is reflected in a high prevalence of stunting during critical periods for development in early childhood (Shonkoff and Phillips, 2000), it is likely that nutrition interventions would also produce significant benefits. The simultaneous delivery of interventions that improve nutrition, preschool education and basic education is likely to be necessary to gain the greatest benefit from the investment in any one of these.

In addition, Vietnamese parents’ reasons for sending, or not sending, their child to preschool may inform the way preschool education is implemented. First, the prominence of costs among the reasons children did not attend preschool sounds a note of caution about the introduction of user-pays models. These may entrench existing disadvantages if children from the most socio-economically disadvantaged families are those least likely to receive the preschool education that could prepare them to succeed at school. Moreover, families living in poverty may choose to save on both preschool and school costs by keeping their young child at home and in the care of an older sibling. In the current study, almost one-in-four of the parents who did not send their child to preschool indicated that this was not necessary because their child was cared for by another child. Such short-term cost savings are likely to be at the price of long-term impairment of both children’s education. Second, Vietnam’s strategy of increasing the uptake of preschool education by making it compulsory is not viable in most low- and middle-income countries which have neither the trained personnel nor the infrastructure necessary to provide universal access to preschool. In such contexts, increases in the preschool attendance of disadvantaged children may only be achieved after there is greater awareness of the role preschool education plays in children’s school readiness. Recognition of its educational benefits was the main reason Vietnamese parents chose to send their children to preschool.

## 5. Conclusion

Each of the four analytic approaches adopted in this research suggest that, even under resource-constrained conditions, preschool education delivered “at scale” can achieve small but meaningful improvements in the size of children’s vocabularies and their mathematics skills, and that these improvements may be detected for at least the following 10 years. Although the magnitude of some between-group differences and dose-effect relationships faded over time, few analyses found evidence of the fade-out of cognitive benefits commonly reported in studies of preschool education in high-income countries. The high cultural value ascribed to learning and the long history of preschool education in Vietnam may limit the generalizability of the current findings to other low- and middle-income countries. Nevertheless, the current findings have a number of policy implications for the implementation of preschool programs in these countries.

## Data availability statement

Publicly available datasets were analyzed in this study. This data can be found at: The data used in this study can be obtained by application to The Young Lives study at the University of Oxford https://www.younglives.org.uk/data.

## Ethics statement

All components of the Young Lives study, from which data of this current study employs, have undergone comprehensive ethical review by the University of Oxford, the Vietnam Union of Science and Technology Associations (VUSTA 2001), and the Hanoi School of Public Health. Written informed consent to participate in this study was provided by the participants’ legal guardian/next of kin.

## Author contributions

PD and JR contributed to all sections of the manuscript. All authors approved the final version of the manuscript.

## Conflict of interest

The authors declare that the research was conducted in the absence of any commercial or financial relationships that could be construed as a potential conflict of interest.

## Publisher’s note

All claims expressed in this article are solely those of the authors and do not necessarily represent those of their affiliated organizations, or those of the publisher, the editors and the reviewers. Any product that may be evaluated in this article, or claim that may be made by its manufacturer, is not guaranteed or endorsed by the publisher.
