# Supplementary material for: Persistence and fading of the cognitive and socio-emotional benefits of preschool education in a low-resource setting: Group differences and dose-dependent associations in longitudinal data from Vietnam
Source: Front Psychol. 2023 Feb 7;14:1065572. doi: 10.3389/fpsyg.2023.1065572 (PMC9942945; doi:10.3389/fpsyg.2023.1065572)
Supplement: Supplementary file 1 [file Table_1.DOCX]

Supplementary Table 1. Descriptive statistics for outcomes measures at 5, 8, 12 and 15 years of age for children who had and not attended preschool

|  | Did not attend preschool  (n = 164) | | |  | | Attended preschool  (n = 1,562) | | |  | |
| --- | --- | --- | --- | --- | --- | --- | --- | --- | --- | --- |
| Child age and outcome variable | Mean | (SD) |  | | Mean | | (SD) | Sig. | |  |
| 5 years |  |  |  | |  | |  |  | |  |
| Numeracy: Rasch score | 281.3 | (49.3) |  | | 304.8 | | (46.6) | ** | |  |
| Receptive vocabulary: Rasch score | 264.3 | (47.3) |  | | 303.5 | | (49.0) | *** | |  |
| Life satisfaction | 3.5 | (1.2) |  | | 4.3 | | (1.5) | ** | |  |
| 8 years |  |  |  | |  | |  |  | |  |
| Mathematics: Rasch score | 289.3 | (12.5) |  | | 300.6 | | (14.6) | ** | |  |
| Receptive vocabulary: Rasch score | 289.2 | (13.8) |  | | 301.3 | | (14.4) | ** | |  |
| Life satisfaction | 3.5 | (1.2) |  | | 4.3 | | (1.5) | ** | |  |
| 12 years |  |  |  | |  | |  |  | |  |
| Mathematics: Percentage correct | 38.5 | (16.3) |  | | 48.7 | | (16.8) | ** | |  |
| Receptive vocabulary: Percentage correct | 72.0 | (13.9) |  | | 77.2 | | (10.3) | * | |  |
| Life satisfaction | 4.9 | (1.8) |  | | 5.5 | | (1.6) | * | |  |
| Self-efficacy | 2.8 | (0.3) |  | | 2.8 | | (0.3) |  | |  |
| Self-esteem | 2.8 | (0.3) |  | | 2.8 | | (0.3) |  | |  |
| Relationships with peers | 2.8 | (0.3) |  | | 2.8 | | (0.3) |  | |  |
| Relationships with parents | 3.2 | (0.4) |  | | 3.3 | | (0.4) |  | |  |
| 15 years |  |  |  | |  | |  |  | |  |
| Mathematics: percentage correct | 37.5 | (20.5) |  | | 47.1 | | (21.3) |  | |  |
| Receptive vocabulary: Percentage correct | 73.4 | (13.5) |  | | 79.2 | | (11.5) | ** | |  |
| Life satisfaction | 4.3 | (1.4) |  | | 4.9 | | (1.4) |  | |  |
| Self-efficacy | 2.8 | (0.3) |  | | 2.9 | | (0.3) |  | |  |
| Self-esteem | 2.8 | (0.3) |  | | 2.8 | | (0.3) |  | |  |
| Relationships with peers | 2.8 | (0.3) |  | | 2.8 | | (0.3) |  | |  |
| Relationships with parents | 3.2 | (0.4) |  | | 3.2 | | (0.4) |  | |  |

* p < .01; ** p < .001
