# Supplementary material for: Persistence and fading of the cognitive and socio-emotional benefits of preschool education in a low-resource setting: Group differences and dose-dependent associations in longitudinal data from Vietnam
Source: Front Psychol. 2023 Feb 7;14:1065572. doi: 10.3389/fpsyg.2023.1065572 (PMC9942945; doi:10.3389/fpsyg.2023.1065572)
Supplement: Supplementary file 2 [file Table_2.docx]

Supplementary Table 2. Summary of findings from hierarchical regression analyses for the relationship between the dose of preschool education children received and the outcome variables for Vietnamese children from families with lower and higher wealth index scores

|  | Step 1: child age (months) | | | |  |  | Step 2: Preschool dose | | | | |  |  |
| --- | --- | --- | --- | --- | --- | --- | --- | --- | --- | --- | --- | --- | --- |
|  |  | | | |  |  |  | | | | |  |  |
| Context, nominal age and outcome | B | SE B | ß | R^2^ | F | Sig. | B | SE B | ß | R^2^ change | | F change | Sig. |
|  |  |  |  |  |  |  |  |  |  |  | |  |  |
| Family with lower wealth |  |  |  |  |  |  |  |  |  | |  |  |  |
| 5 years |  |  |  |  |  |  |  |  |  | |  |  |  |
| Receptive vocabulary | 2.513 | .554 | .184 | .034 | 20.6 | ** | .023 | .004 | .212 | | .042 | 26.7 | ** |
| Numeracy | 2.077 | .534 | .150 | .022 | 15.2 | ** | -.014 | .005 | -.115 | | .012 | 8.3 | * |
| Life satisfaction | 0.001 | .014 | .003 | < .001 | < 0.1 | ns | .001 | <.001 | .177 | | .029 | 21.3 | ** |
| 8 years |  |  |  |  |  |  |  |  |  | |  |  |  |
| Receptive vocabulary | 0.831 | .141 | .322 | .048 | 34.4 | ** | .009 | .001 | .287 | | .077 | 59.7 | ** |
| Mathematics | 0.919 | .145 | .241 | .061 | 45.1 | ** | .001 | .001 | .026 | | .001 | 0.5 | ns |
| Life satisfaction | -0.066 | .038 | -.064 | .004 | 2.9 | ns | -.001 | <.001 | -.094 | | .008 | 5.9 | ns |
| 12 years |  |  |  |  |  |  |  |  |  | |  |  |  |
| Receptive vocabulary | .031 | .129 | .009 | <.001 | 0.1 | ns | .005 | .001 | .184 | | .032 | 22.9 | ** |
| Mathematics | .375 | .171 | .083 | .007 | 4.8 | ns | .004 | .001 | .109 | | .011 | 7.7 | * |
| Life satisfaction | -.035 | .018 | -.073 | .005 | 3.8 | ns | .001 | <.001 | .173 | | .028 | 20.4 | ** |
| Self-efficacy | -.003 | .004 | -.036 | .001 | 0.9 | ns | <.001 | <.001 | .069 | | .004 | 3.1 | ns |
| Self-esteem | -.002 | .004 | -.020 | <.001 | 0.3 | ns | <.001 | <.001 | .072 | | .005 | 3.4 | ns |
| Peer relations | -.005 | .004 | -.054 | .002 | 1.7 | ns | <.001 | <.001 | .020 | | <.001 | 0.3 | ns |
| Parent relations | .001 | .004 | .005 | <.001 | <0.1 | ns | <.001 | <.001 | .007 | | <.001 | <0.1 | ns |
| 15 years |  |  |  |  |  |  |  |  |  | |  |  |  |
| Receptive vocabulary | .009 | .140 | .003 | <.001 | <0.1 | ns | .007 | .001 | .100 | | .041 | 30.0 | ns |
| Mathematics | .512 | .208 | .093 | .009 | 6.0 | ns | .003 | .002 | .056 | | .003 | 2.0 | ns |
| Life satisfaction | -.014 | .015 | -.036 | .001 | 0.9 | ns | <.001 | <.001 | .088 | | .007 | 5.2 | ns |
| Self-efficacy | .002 | .003 | .020 | <.001 | 0.2 | ns | <.001 | <.001 | -.071 | | .005 | 3.3 | ns |
| Self-esteem | .004 | .003 | .049 | .002 | 1.7 | ns | <.001 | <.001 | -.010 | | <.001 | 0.1 | ns |
| Peer relations | .002 | .003 | .027 | .001 | 0.5 | ns | <.001 | <.001 | -.005 | | <.001 | <.001 | ns |
| Parent relations | .001 | .005 | .005 | <.001 | <0.1 | ns | <.001 | <.001 | -.118 | | .013 | 9.2 | * |
| Family with higher wealth |  |  |  |  |  |  |  |  |  | |  |  |  |
| 5 years |  |  |  |  |  |  |  |  |  | |  |  |  |
| Receptive vocabulary | 1.787 | 0.521 | .126 | .016 | 11.8 | ** | .039 | .003 | .413 | | .161 | 142.3 | ** |
| Numeracy | 1.790 | 0.445 | .141 | .020 | 16.2 | ** | .017 | .003 | .202 | | .039 | 32.5 | ** |
| Life satisfaction | -0.008 | .013 | -.020 | <.001 | 0.3 | ns | <.001 | <.001 | .051 | | .002 | 2.0 | ns |
| 8 years |  |  |  |  |  |  |  |  |  | |  |  |  |
| Receptive vocabulary | 0.665 | .132 | .180 | .032 | 25.5 | ** | .005 | .001 | .187 | | .033 | 27.2 | ** |
| Mathematics | 1.020 | .128 | .269 | .072 | 63.4 | ** | .006 | .001 | .204 | | .040 | 36.3 | ** |
| Life satisfaction | -.053 | .019 | -.096 | .009 | 7.7 | * | <.001 | <.001 | -.025 | | .001 | 0.5 | ns |
| 12 years |  |  |  |  |  |  |  |  |  | |  |  |  |
| Receptive vocabulary | .132 | .088 | .053 | .003 | 2.2 | ns | .003 | .001 | .176 | | .029 | 23.5 | ** |
| Mathematics | .012 | .165 | .003 | <.001 | <0.1 | ns | .006 | .167 | .197 | | .037 | 29.3 | ** |
| Life satisfaction | -.005 | .015 | -.012 | <.001 | 0.1 | ns | <.001 | <.001 | -.014 | | <.001 | 0.1 | ns |
| Self-efficacy | <.001 | .003 | -.001 | .004 | <0.1 | ns | <.001 | <.001 | -.038 | | .001 | 1.0 | ns |
| Self-esteem | .001 | .003 | .008 | .002 | 1.8 | ns | <.001 | <.001 | -.056 | | <.001 | 0.1 | ns |
| Peer relations | -.002 | .004 | -.007 | <.001 | 0.4 | ns | <.001 | <.001 | -.059 | | .003 | 2.6 | ns |
| Parent relations | -.002 | .004 | -.022 | <.001 | 0.4 | ns | <.001 | <.001 | -.059 | | .003 | 2.6 | ns |
| 15 years |  |  |  |  |  |  |  |  |  | |  |  |  |
| Receptive vocabulary | .104 | .106 | .034 | .001 | 0.9 | ns | .002 | .001 | .100 | | .009 | 7.7 | ns |
| Mathematics | -.067 | .213 | -.072 | <.001 | 0.1 | ns | .011 | .001 | .280 | | .075 | 64.7 | ** |
| Life satisfaction | .008 | .014 | .021 | <.001 | 0.4 | ns | <.001 | <.001 | .009 | | <.001 | 0.1 | ns |
| Self-efficacy | -.002 | .003 | -.022 | <.001 | 0.4 | ns | <.001 | <.001 | -.146 | | .020 | 16.4 | ns |
| Self-esteem | .001 | .003 | .043 | <.001 | 0.1 | ns | <.001 | <.001 | -.139 | | .018 | 14.5 | ** |
| Peer relations | .003 | .003 | .037 | .001 | 1.0 | ns | <.001 | <.001 | -.131 | | .016 | 12.8 | ** |
| Parent relations | .002 | .004 | .013 | <.001 | 0.1 | ns | <.001 | <.001 | -.115 | | .013 | 10.1 | * |
|  |  |  |  |  |  |  |  |  |  | |  |  |  |

* p < .01; ** p < .001; ns: p > .01
