# Supplementary material for: Persistence and fading of the cognitive and socio-emotional benefits of preschool education in a low-resource setting: Group differences and dose-dependent associations in longitudinal data from Vietnam
Source: Front Psychol. 2023 Feb 7;14:1065572. doi: 10.3389/fpsyg.2023.1065572 (PMC9942945; doi:10.3389/fpsyg.2023.1065572)
Supplement: Supplementary file 3 [file Table_3.DOCX]

Supplementary Table 3. Demographic differences between child who were and were not enrolled in school at 15 years of age

|  | Not enrolled in school  (n = 293) | | | | | | Enrolled in school  (n = 1,367) | | | | | | | | | |  |  |  |
| --- | --- | --- | --- | --- | --- | --- | --- | --- | --- | --- | --- | --- | --- | --- | --- | --- | --- | --- | --- |
| Variable | % | | M | | (SD) | | % | |  | M | | | SD | | | Statistic | |  |  |
| Rural residence | | 92.5 | |  | |  | | 78.8 | | |  |  | | |  | 𝛘^2^_(1)_ = 29.7** | | |  |
| Male | | 70.2 | |  | |  | | 44.2 | | |  |  | |  | | 𝛘^2^_(1)_ = 1670.4** | | | |
| Family wealth index^ | |  | | 0.661 | | (0.415) | |  | | |  | 0.727 | | (0.130) | | t_(443.6)_ = -8.3^#^** | | | |
| Child age | |  | | 183.3 | | (3.4) | |  | | |  | 182.3 | | (3.7) | | t_(507.6)_ = -4.9^#^** | | | |

^ The Wealth Index was calculated from information concerning quality of housing, access to basic services and ownership of consumer durables (Young Lives, 2002). Scores can range between 0 and 1, with higher scores indicating higher wealth.

** p < .001

^#^ Equal variance not assumed
