# Supplementary material for: Persistence and fading of the cognitive and socio-emotional benefits of preschool education in a low-resource setting: Group differences and dose-dependent associations in longitudinal data from Vietnam
Source: Front Psychol. 2023 Feb 7;14:1065572. doi: 10.3389/fpsyg.2023.1065572 (PMC9942945; doi:10.3389/fpsyg.2023.1065572)
Supplement: Supplementary file 4 [file Table_4.DOCX]

Supplementary Table 4. Associations between the dose of preschool education received by Vietnamese children who we no longer enrolled in school and 15 years and their cognitive and socio-emotional outcomes at 15 years after controlling for individual differences in child age and family wealth index

| Outcome | R^2^ change | |  |
| --- | --- | --- | --- |
| Cognitive | |  |  |
| Receptive vocabulary | | .008 |  |
| Quantity/Mathematics | | .025 |  |
| Socio-emotional | |  |  |
| Life satisfaction | | .014 |  |
| Self-efficacy | | .009 |  |
| Self-esteem | | .007 |  |
| Relationship with peers | | .004 |  |
| Relationship with parents | | .043* |  |

* p < .01
