# Supplementary material for: Persistence and fading of the cognitive and socio-emotional benefits of preschool education in a low-resource setting: Group differences and dose-dependent associations in longitudinal data from Vietnam
Source: Front Psychol. 2023 Feb 7;14:1065572. doi: 10.3389/fpsyg.2023.1065572 (PMC9942945; doi:10.3389/fpsyg.2023.1065572)
Supplement: Supplementary file 5 [file Table_5.docx]

Supplementary Table 5. Descriptive statistics for outcomes measures at 5, 8, 12 and 15 years of age for rural children who had different doses of preschool education and urban children who had a high dose of preschool education

|  | Rural | | | | | | | | | | |  | Urban | |
| --- | --- | --- | --- | --- | --- | --- | --- | --- | --- | --- | --- | --- | --- | --- |
|  | No preschool  (n = 152) | |  | Less than 1000 hours preschool  (n = 426) | |  | 1000-2999 hours preschool  (n = 411) | |  | 3000 hours or more preschool  (n = 373) | |  | 3000 hours or more preschool  (n = 337) | |
| Child age and outcome variable | Mean | (SD) |  | Mean | (SD) |  | Mean | (SD) |  | Mean | (SD) |  | Mean | (SD) |
| 5 years |  |  |  |  |  |  |  |  |  |  |  |  |  |  |
| Numeracy: Rasch score | 267.7 | (60.1) |  | 298.4 | (57.7) |  | 293.9 | (46.0) |  | 304.4 | (39.9) |  | 321.5 | (36.5) |
| Receptive vocabulary: Rasch score | 254.6 | (41.6) |  | 278.7 | (44.8) |  | 288.5 | (47.8) |  | 312.6 | (43.5) |  | 341.6 | (39.3) |
| Life satisfaction | 3.5 | (1.3) |  | 3.9 | (1.4) |  | 4.2 | (1.3) |  | 4.8 | (1.6) |  | 4.5 | (1.3) |
| 8 years |  |  |  |  |  |  |  |  |  |  |  |  |  |  |
| Mathematics: Rasch score | 288.3 | (12.1) |  | 296.0 | (12.4) |  | 299.1 | (15.1) |  | 302.0 | (13.9) |  | 309.0 | (14.8) |
| Receptive vocabulary: Rasch score | 286.2 | (14.4) |  | 294.8 | (13.0) |  | 298.8 | (14.0) |  | 306.2 | (13.4) |  | 308.0 | (14.3) |
| Life satisfaction | 5.5 | (2.4) |  | 6.0 | (2.2) |  | 5.8 | (2.2) |  | 5.9 | (2.1) |  | 6.3 | (2.1) |
| 12 years |  |  |  |  |  |  |  |  |  |  |  |  |  |  |
| Mathematics: Percentage correct | 37.1 | (15.9) |  | 43.9 | (15.2) |  | 46.2 | (16.4) |  | 54.0 | (17.3) |  | 53.1 | (15.0) |
| Receptive vocabulary: Percentage correct | 69.6 | (15.0) |  | 74.8 | (12.2) |  | 75.8 | (10.8) |  | 79.6 | (7.6) |  | 79.9 | (8.9) |
| Life satisfaction | 4.9 | (1.7) |  | 5.3 | (1.6) |  | 5.5 | (1.5) |  | 5.7 | (1.5) |  | 5.5 | (1.6) |
| Self-efficacy | 2.8 | (0.3) |  | 2.8 | (0.3) |  | 2.9 | (0.3) |  | 2.8 | (0.3) |  | 2.7 | (0.3) |
| Self-esteem | 2.8 | (0.3) |  | 2.7 | (0.3) |  | 2.8 | (0.3) |  | 2.8 | (0.3) |  | 2.7 | (0.3) |
| Relationships with peers | 2.8 | (0.3) |  | 2.8 | (0.3) |  | 2.8 | (0.3) |  | 2.8 | (0.3) |  | 2.7 | (0.3) |
| Relationships with parents | 3.2 | (0.4) |  | 3.3 | (0.4) |  | 3.2 | (0.4) |  | 3.3 | (0.4) |  | 3.2 | (0.4) |
| 15 years |  |  |  |  |  |  |  |  |  |  |  |  |  |  |
| Mathematics: percentage correct | 35.4 | (19.1) |  | 40.3 | (19.2) |  | 43.9 | (20.5) |  | 50.3 | (21.5) |  | 58.3 | (20.2) |
| Receptive vocabulary: Percentage correct | 70.6 | (16.1) |  | 75.5 | (12.8) |  | 77.8 | (12.2) |  | 83.6 | (9.9) |  | 79.5 | (10.5) |
| Life satisfaction | 4.4 | (1.4) |  | 4.7 | (1.4) |  | 4.9 | (1.4) |  | 5.2 | (1.3) |  | 4.9 | (1.4) |
| Self-efficacy | 2.8 | (0.3) |  | 2.9 | (0.3) |  | 2.9 | (0.3) |  | 2.9 | (0.3) |  | 2.9 | (0.3) |
| Self-esteem | 2.8 | (0.3) |  | 2.8 | (0.3) |  | 2.8 | (0.3) |  | 2.8 | (0.3) |  | 2.7 | (0.3) |
| Relationships with peers | 2.8 | (0.3) |  | 2.8 | (0.3) |  | 2.8 | (0.3) |  | 2.8 | (0.3) |  | 2.8 | (0.3) |
| Relationships with parents | 3.2 | (0.4) |  | 3.3 | (0.4) |  | 3.2 | (0.4) |  | 3.2 | (0.4) |  | 3.2 | (0.5) |

* p < .01; ** p < .001
